# Parallel evolution of senescence in annual fishes in response to extrinsic mortality

**DOI:** 10.1186/1471-2148-13-77

**Published:** 2013-04-03

**Authors:** Eva Terzibasi Tozzini, Alexander Dorn, Enoch Ng’oma, Matej Polačik, Radim Blažek, Kathrin Reichwald, Andreas Petzold, Brian Watters, Martin Reichard, Alessandro Cellerino

**Affiliations:** 1 Fritz-Lipmann Institute for Age Research, Leibniz Institute, Jena, Germany; 2 Scuola Normale Superiore, Pisa, Italy; 3 Institute of Vertebrate Biology Academy of Sciences of the Czech Republic, Brno, Czech Republic; 4 36141 Parkwood Drive, Nanaimo, British Columbia, Canada

**Keywords:** Ageing theory, Life history, Trade off, *Nothobranchius*, Lipofuscin

## Abstract

**Background:**

Early evolutionary theories of aging predict that populations which experience low extrinsic mortality evolve a retarded onset of senescence. Experimental support for this theory in vertebrates is scarce, in part for the difficulty of quantifying extrinsic mortality and its condition- and density-dependent components that –when considered- can lead to predictions markedly different to those of the “classical” theories. Here, we study annual fish of the genus *Nothobranchius* whose maximum lifespan is dictated by the duration of the water bodies they inhabit. Different populations of annual fish do not experience different strengths of extrinsic mortality throughout their life span, but are subject to differential timing (and predictability) of a sudden habitat cessation. In this respect, our study allows testing how aging evolves in natural environments when populations vary in the prospect of survival, but condition-dependent survival has a limited effect. We use 10 *Nothobranchius* populations from seasonal pools that differ in their duration to test how this parameter affects longevity and aging in two independent clades of these annual fishes.

**Results:**

We found that replicated populations from a dry region showed markedly shorter captive lifespan than populations from a humid region. Shorter lifespan correlated with accelerated accumulation of lipofuscin (an established age marker) in both clades. Analysis of wild individuals confirmed that fish from drier habitats accumulate lipofuscin faster also under natural conditions. This indicates faster physiological deterioration in shorter-lived populations.

**Conclusions:**

Our data provide a strong quantitative example of how extrinsic mortality can shape evolution of senescence in a vertebrate clade. *Nothobranchius* is emerging as a genomic model species. The characterization of pairs of closely related species with different longevities should provide a powerful paradigm for the identification of genetic variations responsible for evolution of senescence in natural populations.

## Background

Despite the identification of single-gene mutations that can retard ageing in laboratory species [1,2] little is known about genetic mechanisms controlling evolution of longevity and senescence under natural conditions, particularly in vertebrates [3]. The influence of extrinsic mortality on evolution of aging is a subject of great theoretical interest, but has received limited experimental tests in vertebrates [4-8]. Evolutionary theories of aging [9-11] indicate that senescence is a result of age-dependent decrease in the force of selection and predict that populations experiencing lower mortality due to external causes evolve slower senescence. Studies of experimental evolution in *Drosophila* have confirmed that experimental modulation of extrinsic mortality can lead to retarded senescence [12].

Despite enormous effort to study senescence in wild populations (reviewed in [13]) our knowledge about evolution of longevity and senescence under natural conditions in vertebrates is very limited [4,8]. This is mainly because only few studies were able to employ replicated populations (e.g. [5,6]). Beside difficulties to obtain data on replicated populations, there are several other reasons for the lack of a better understanding of senescence in the wild. There is a need for resighting or recapture of the same individuals, the long lifespan of most vertebrates, the recent elucidation of more complicated predictions regarding evolution of aging, the difficulty in quantifying extrinsic mortality and several possibilities how exactly to measure senescence rate.

The relationship between extrinsic mortality and senescence may be more complex than suggested by early theory [9,10,14]. For example, age classes may vary in their susceptibility to extrinsic mortality [15] or increases in extrinsic mortality rate may be accompanied by decreases in population density and increases in resource availability to survivors [16]. When these additional variables are included, model predictions are affected and increased extrinsic mortality may result in either accelerated senescence, retarded senescence, or no overall change in patterns of senescence. Further, another important issue is how mortality risk is realized. For example, if survival in the wild is condition-dependent [17], higher extrinsic mortality rate can lead to slower rather than more rapid senescence, despite the fact that the same genotypes evolve according to classical predictions when mortality is applied randomly [18]. It corroborates earlier evidence that it is important to consider how mortality is applied in the selection experiments on *Drosophila*[19,20].

In our study system, different populations of annual fish do not experience different strengths of extrinsic mortality throughout their life span (such as from contrasting predation pressure), but are subject to differential timing (and predictability) of a sudden habitat cessation. In this respect, our study allows to test how aging evolves in natural environments when condition dependent survival has a limited effect.

Up to date, the only study of evolution of senescence in replicated natural populations of vertebrates provided outcomes challenging several points of evolutionary theory of ageing. In that study a small tropical fish, guppy (*Poecilia reticulata*) was used with populations experiencing high- and low-predation conditions and found, contrary to expectation, that populations from high-predation environments had slower senescence than low-predation populations [6,21], possibly because predation-dependent mortality was condition-dependent.

Annual fish of the genus *Nothobranchius* offer a remarkable system to study the effects of extrinsic mortality on evolution of aging due to their naturally short lifespan and strictly nonoverlapping generations [22]. *Nothobranchius* fish inhabit temporary pools in Eastern Africa that are flooded during intense monsoonal precipitation, leading to synchronous hatching of eggs [23]. This ensures that all fish in a given pool are of the same age and that the upper limit of longevity is set by the desiccation of the pool. In this respect, different populations of annual fish do not experience different strengths of extrinsic mortality throughout their life span. The age-mortality relationship is almost rectangular and extrinsic mortality abruptly reaches 100% in all populations, but the timing of this increase differs. Therefore, these taxa allow to test how aging evolves when the survival abruptly drops to zero after different periods of time. Importantly, these fish retain their short lifespan when reared in captivity and express typical vertebrate aging markers [24-34]. A substantial heterogeneity in the precipitation pattern and hence the time window for the completion of the life cycle is observed across the distribution range of the genus *Nothobranchius*[22]. Therefore, fish from more arid habitats are predicted to experience a shorter maximum lifespan.

**Figure 1:**
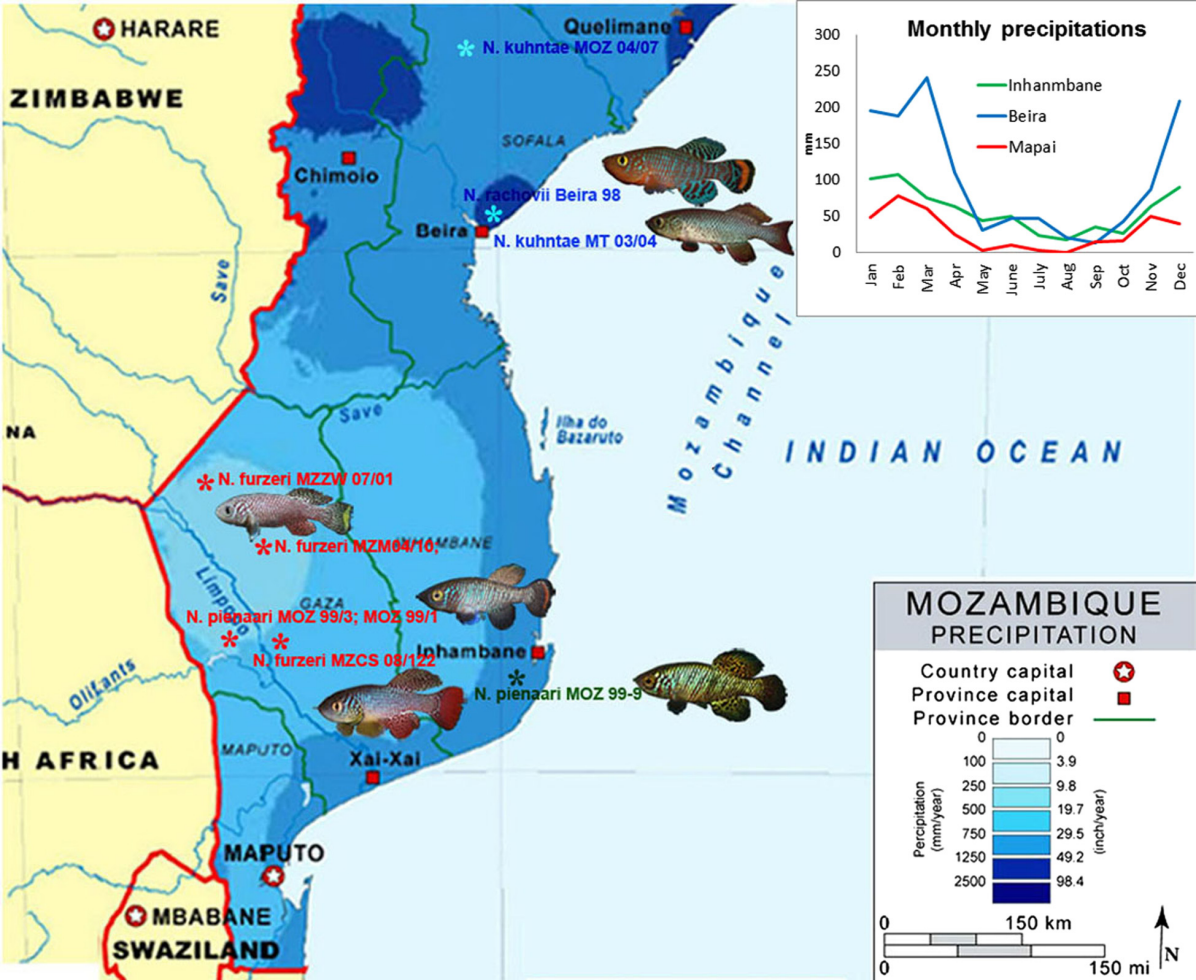
**Distribution map for the *Nothobranchius *populations used in the present study.** Physical map with annual precipitations was obtained from Stock Map Agency (http://www.stockmapagency.com). Collection points are indicated with asterisks and are color-coded: red indicates semi-arid green intermediate and blue humid habitats. Please note that the collection points of *N*. *rachovii* and two of the populations of *N*. *kuhntae* are in Beira and are collectively represented by a single asterisk. Inset shows monthly precipitations in Beira, Inhambane and Mapai as examples of humid, intermediate and semi-arid regions.

Here, we test whether differences in habitat duration led to the evolution of a different rate of senescence in this clade of annual fish. We studied *Nothobranchius* populations from Southern and Central Mozambique because a striking aridity cline is present in this region. The inland region in southern Mozambique receives total annual precipitation as low as 400 mm/year, in contrast to coastal areas near the city of Beira where total annual precipitation is about 1200 mm/year (Figure 1). Two independent evolutionary lineages of *Nothobranchius* are found in this area: *N*. *furzeri and N*. *kuhntae* belong to one lineage while *N*. *rachovii* and *N*. *pienaari* belong to another lineage [35-37]. For each of these two evolutionary lineages, one species originates from the semi-arid inland habitat (*N*. *furzeri* and *N*. *pienaari*, respectively) and the other species from the humid coastal area (*N*. *kuhntae* and *N*. *rachovii*, respectively). This enables us to investigate whether there is parallel evolution of senescence in two independent evolutionary lineages (Figure 1). As hatching is synchronized across dry and humid regions due to periods of intensive cyclone-based precipitation over large areas, fish across our study area are of the same age [23]. Therefore, the critical factor in determining the maximum window of survival in the wild is the calendar date of habitat desiccation.

Importantly, fish of the genus *Nothobranchius* are emerging as model laboratory species for genetic studies of ageing. There is a growing number of genetic/genomic resources available [38-40] and quantitative trait loci controlling longevity were detected in crosses of laboratory populations [41].

## Results

### Habitat desiccation

We used two complementary approaches to directly confirm that pools in more humid regions hold water for longer periods of time than pools in more arid regions – direct observation and the use of dataloggers.

At the beginning of the dry season in 2011 and 2012, we visited sites where presence of *Nothobranchius* fish was detected in previous years (Reichard et al. 2009; Dorn et al. 2011) and recorded whether particular habitats had desiccated or not. There was a clear difference between regions; in ‘semi-arid’ regions most sites were dry, whereas in ‘humid’ regions most were filled (Table 1, Fisher’s exact test: p = 0.0016 for 2011, p = 0.0006 for 2012).

**Table 1:** The number of sites with water and desiccated and their percentage from the total number of sites visited in 2011 and 2012

	**2011**				**2012**			
	**water**		**dry**		**water**		**dry**	
region	N	%	N	%	N	%	N	%
humid	15	**75**	5	**25**	18	**75**	6	**25**
semi-dry	23	**33**	47	**67**	11	**29**	27	**71**

**Figure 2:**
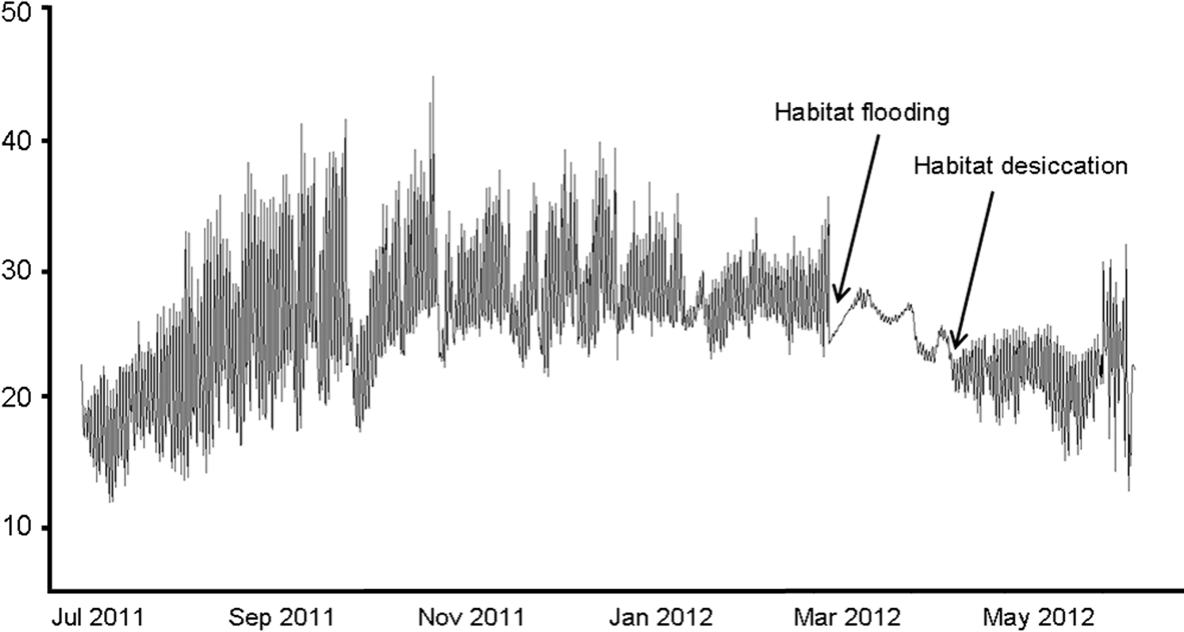
**Temperature fluctuations (logging every 3 hours) at site MZCS 207 (semi-arid region) from a period between 29 June 2011 to 23 May 2012, with a single period of habitat duration.** The estimated time of filling and drying of the pond are indicated.

The time of the pool desiccation was estimated using waterproof dataloggers that were left in habitats during previous visits (Table 1). Filling and desiccation of the pool was indicated by abrupt decrease and increase of amplitude of daily temperature fluctuation (Figure 2). In 2011, dataloggers were placed after the start of the rain season (13 February to 12 March) and recovered at a later timepoint, 6 out of 7 loggers and 0 out of 2 loggers that had been recovered between 7 and 11 July were dry in semi-arid region and humid region, respectively. In 2012, dataloggers placed in 2011 were recovered and therefore detected also the filling of the ponds. Ten out of 12 loggers and 1 out of 3 loggers that had been recovered (25 May to 7 June 2012) were already dry in semi-arid region and humid region, respectively.

In 2011, the mean date of pool desiccation estimated from the logged temperature fluctuations in semi-arid region was 25 April (s.e. = 12 days, range 7 March – 24 June, n = 6 sites). In 2012, the mean date of pool desiccation in semi-arid region was 29 March (s.e. = 17 days, range 7 February – 7 June n = 7 sites; 3 sites with ambiguous reading excluded). For 2012, loggers clearly demonstrated synchronous flooding of all sites, with flooding on 15 January 2012 apparent at all sites except for one site in semi-arid region which was not flooded and remained dry throughout the wet season 2012 (despite *Nothobranchius* presence in previous years). This shows that, during this season, habitats in semi-arid region were flooded for a mean of 73 days (=10 weeks; median 76 days), while habitats in humid region were flooded for at least 142 days (=20 weeks), but likely longer (minimum estimate taken from time of logger recovery when sites were still flooded).

### Captive longevity of *Nothobranchius* populations

**Figure 3:**
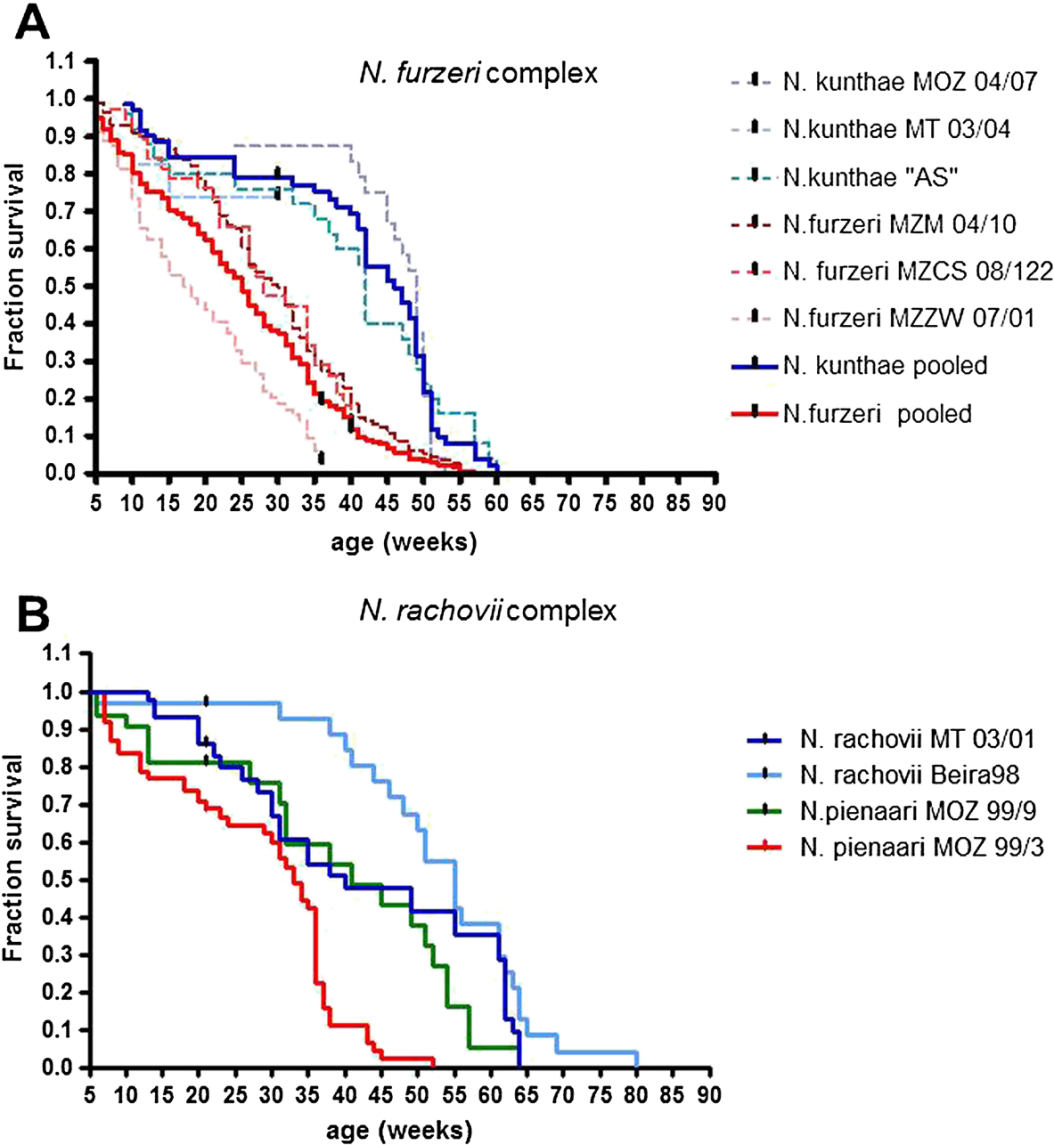
**Age-dependent survival of *N. furzeri *species complex and *N. rachovii *species complex.** (**A**) Survivorship of *N. furzeri* MZZW 07/01 (pink broken line n = 124), *N. furzeri* MZM 04/10 (red broken line, n = 113), *N. furzeri* MZCS 08/122 (brown broken line, n = 33), *N. kuhntae* MT-03/04 (light blue broken line n = 23; censored at age 33 weeks due to disease outbreak), *N. kuhntae* “aquarium strain” (blue broken line, n = 25) and *N. kuhntae* MOZ 04/07 (dark blue broken line, n = 24). Pooled survivorship of *N. furzeri* (n = 223) is shown in solid red and the survivorship of pooled *N. kuhntae* (n = 72) is shown in solid blue. The difference in the survivorship between the two pooled groups is highly significant (Log-Rank test, p < 0.0001). For descriptive statistics and pair wise comparisons see Additional file 1: Table S2, Additional file 2: Table S3 – (**B**) Survivorship of *N. pienaari* MOZ 99/3 (red line, n = 61). *N. pienaari* MOZ 99/9 (green line, n = 31), *N. rachovii* Beira 98 (light blue line, n = 34) and *N. rachovii* MT 03/01 (blue line, n = 43). For descriptive statistics and pair-wise comparisons see Additional file 4: Table S4, Additional file 5: Table S5.

In total, we recorded captive lifespan of 10 different populations from four different *Nothobranchius* species belonging to two evolutionary lineages (*N*. *furzeri* clade and *N*. *rachovii* clade) and two habitat types (semi-arid and humid) (Table 2). We analyzed the lifespan of three independent captive populations of *N*. *furzeri* (semi-arid habitat) for which the collection point is known. These populations span the entire distribution range of the species. For comparisons, we measured lifespan of three independent captive populations of the closely related species *N*. *kuhntae* (humid habitats) for which the collection point is known (Figure 3). One of the *N*. *kuhntae* populations could be followed only until age 33 weeks due to disease outbreak in their tank, but was included in the analysis as censored at age > 33 weeks. The longevity of each of the three *N*. *furzeri* populations (Figure 3A, red broken lines) was shorter than any of the three *N*. *kuhntae* populations (Figure 3A, blue broken lines). It should be remarked, that none of these wild-derived *N*. *furzeri* populations replicated the extremely short lifespan (3 months) observed in the laboratory strain GRZ [42]. Median survival of the pooled *N*. *kuhntae* population (Figure 3A, blue solid line) was almost doubled with respect to the pooled *N*. *furzeri* populations (Figure 3A, red broken line) (47 weeks vs. 24 weeks; Additional file 1: Table S2, log rank test, p < 0.0001) and 10% survivorship was larger by 38% (51 weeks vs. 37 weeks; Additional file 1: Table S2). Out of nine pairwise comparisons between *N*. *furzeri* and *N*. *kuhntae* (Additional file 2: Table S3), 7 were significant (log rank test p < 0.001). Only the two comparisons involving the censored *N*. *kuhntae* population were not significant (long-rank test, p = 0.08). Further, the shape of the curves is apparently different in *N*. *furzeri* and *N*. *kunthae*. Analysis of age-specific mortality suggests that when mortality is fitted by the Gompertz function ***ae***^***bt***^, differences in longevity are rather accounted for by large differences in initial mortality **a** than in the rate of aging **b** (Additional file 3: Figure S1). This datum should be taken with caution, however, due to a small sample size in *N*. *kuhntae*.

**Table 2:** Summary of the populations used in the study

**Population**	**Species**	**Habitat**	**Year of collection**	**Generation**	**Sample size**	**Median life span**	**10% survivorship**
MZM 04/10	*N. furzeri*	semi-arid	2004	~F10	113	29 weeks	40 weeks
MZZW 07/01	*N. furzeri*	semi-arid	2007	F3	124	17.5 weeks	33 weeks
MZCS 08/122	*N.furzeri*	semi-arid	2010	F2	33	28 weeks	40 weeks
MT 03/02	N.kunthae***	humid	2003	n.a.**	23	n.a.*	n.a.*
MOZ 04/07	*N.kuhntae*	humid	2004	n.a.**	25	42 weeks	57 weeks
AS	N.kuhntae***	humid	n.a. (aquarium strain)	n.a.**	24	49 weeks	50 weeks
MOZ 99/3	*N. pienaari*	semi-arid	1999	n.a.**	61	33 weeks	43 weeks
MOZ 99/9	*N. pienaari*	Intermediate	1999	n.a.**	31	41 weeks	55 weeks
MT 03/01	N. rachovii***	humid	2003	n.a.**	43	40 weeks	63 weeks
BEIRA 98	N. rachovii***	humid	1998	n.a.**	34	55 weeks	65 weeks

*censored at age 33 weeks when survival was 74%.

** not collected by the authors, but commercially available.

*** syntopic species in Beira.

In the *N*. *rachovii* clade, longevity was measured in two independent populations of *N*. *rachovii* (both from coastal humid habitat, sympatric with *N*. *kuhntae*, Figure 3B, light and dark blue line) and populations of *N*. *pienaari*. One *N*. *pienaari* population came from semi-arid habitat (sympatric with *N*. *furzeri* Figure 3B, red line). The second *N*. *pienaari* population originated from an intermediate coastal habitat (outside of the *N*. *furzeri* distribution range, Figure 3B, green line). The longevity of the semi-arid *N*. *pienaari* population was shorter than that of each of the two *N*. *rachovii* populations. Median survival of the pooled *N*. *rachovii* populations was 54% larger than the semi-arid *N*. *pienaari* population (51 weeks vs. 33 weeks; Additional file 4: Table S4, log-rank p < 0.001) and the 10% survivorship was 66% larger (63 weeks vs. 38 weeks, Additional file 4: Table S4). The longevity of the *N*. *pienaari* population from intermediate habitat was longer than *N*. *pienaari* from semi-arid habitat (log-rank, p < 0.001, Additional file 5: Table S5) and shorter than that of the two *N*. *rachovii* populations pooled (log-rank, p = 0.001, Additional file 5: Table S5).

### Accumulation of lipofuscin in captive and wild *Nothobranchius* populations

To corroborate that a shorter lifespan in populations from semi-arid region was the result of a faster rate of physiological deterioration, we analyzed a well-accepted age marker, lipofuscin, an auto-fluorescent pigment that accumulates over time in a large variety of organisms [32,43-45]. In invertebrates, association between neurolipofuscin deposition and natural mortality is a phylogenetically and environmentally widespread phenomenon, making it a unique integrative marker of aging [46] and lipofuscin is a predictor of individual longevity in *C*. *elegans*[47]. The association between lipofuscin and rate of ageing is less established in vertebrates, but it still represents the best age marker available, and should provide a consistent estimate for a taxonomically constrained analysis like ours.

Lipofuscin accumulation was analyzed at age 21 weeks in the liver and brain of two populations from each habitat within each clade. For *N*. *furzeri* and *N*. *kuhntae*, we analyzed populations for which lifespan data are available (Table 2: MZZW 07/01 and MZM 04/10 for *N*. *furzeri*; MT 03/02 and MOZ 04/07 for *N*. *kuhntae*) . For *N*. *rachovii* also, we analyzed populations for which lifespan data are available (Table 2: Beira 98 and MT 03/01). For *N*. *pienaari*, we analyzed the populations MOZ 99/3, for which lifespan data are available (Table 2), and the population MOZ 99/1 (semi-arid habitat) that we obtained in too small numbers and therefore was used for histological analysis only.

**Figure 4:**
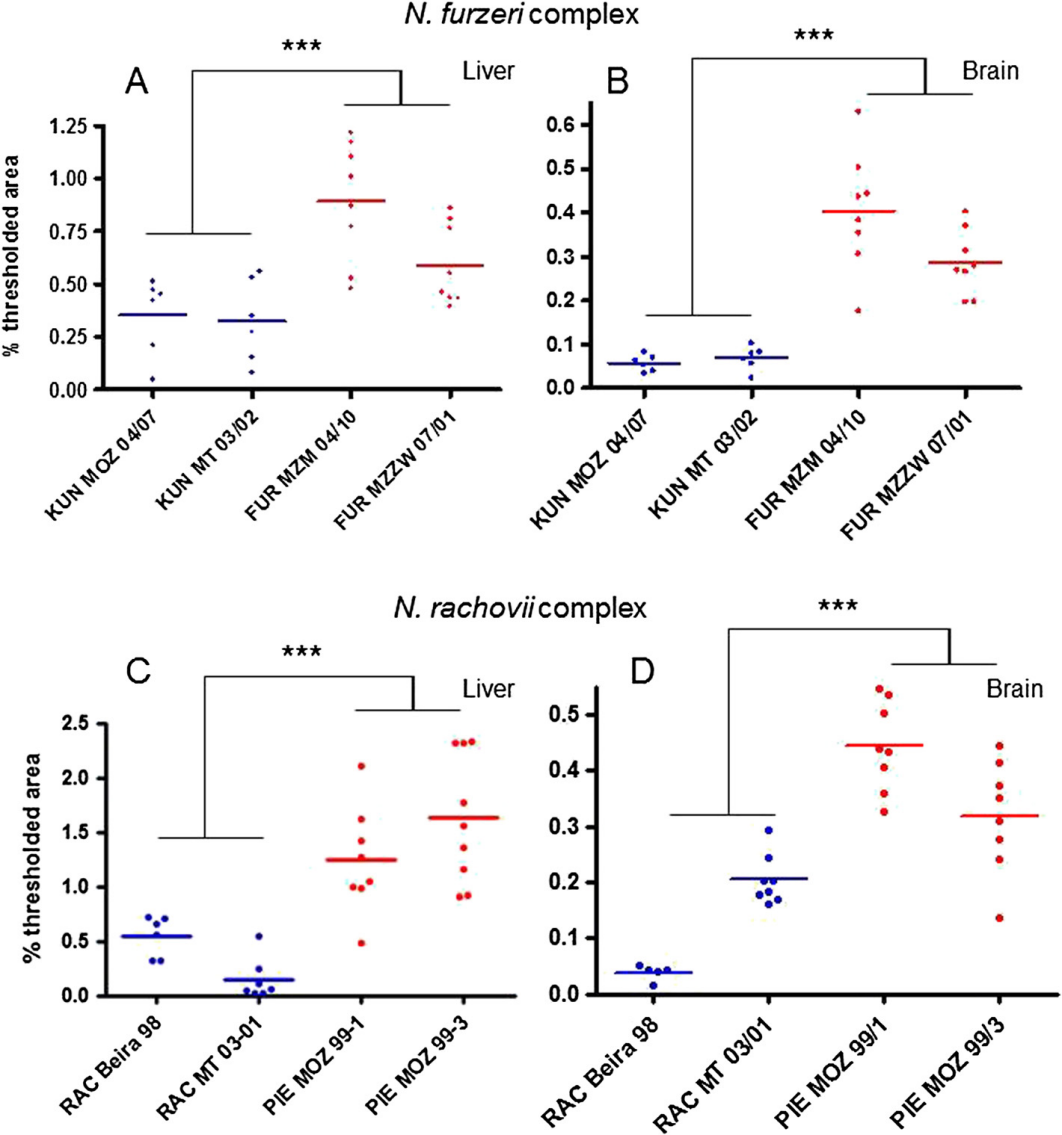
**Expression of lipofuscin in captive individuals of *N. furzeri *species complex and *N. rachovii *species complex.** (**A**) *N. furzeri* complex, expression of lipofuscin in the liver at age 21 weeks. (**B**) *N. furzeri* complex, expression of lipofuscin in the brain at age 21 weeks. (**C**) *N. rachovii* complex, expression of lipofuscin in the liver at age 21 weeks. (**D**) *N. rachovii* complex, expression of lipofuscin in the liver at age 21 weeks. FUR = *N. furzeri*, KUN = *N. kuhntae*, RAC = *N. rachovii*, PIE = *N. pienaari*. In all graphs, red points refer to populations from semi-arid habitats and blue point to populations from humid habitats. Lipofuscin is quantified as percentage of pixels in the image that are brighter than a fixed fluorescence threshold. *** = p < 0.001,Mann Whithney’s U-test. For pairwise comparisons see Additional file 6: Table S6, Additional file 7: Table S7, Additional file 8: Table S8 Additional file 9: Table S9.

In both clades, the populations from the semi-arid habitat had higher lipofuscin loads than the populations from the humid habitat of the same chronological age (Figure 4, for pair-wise comparisons see Additional file 6: Tables S6 Additional file 7: Table S7, Additional file 8: Table S8, Additional file 9: Table S9).

**Figure 5:**
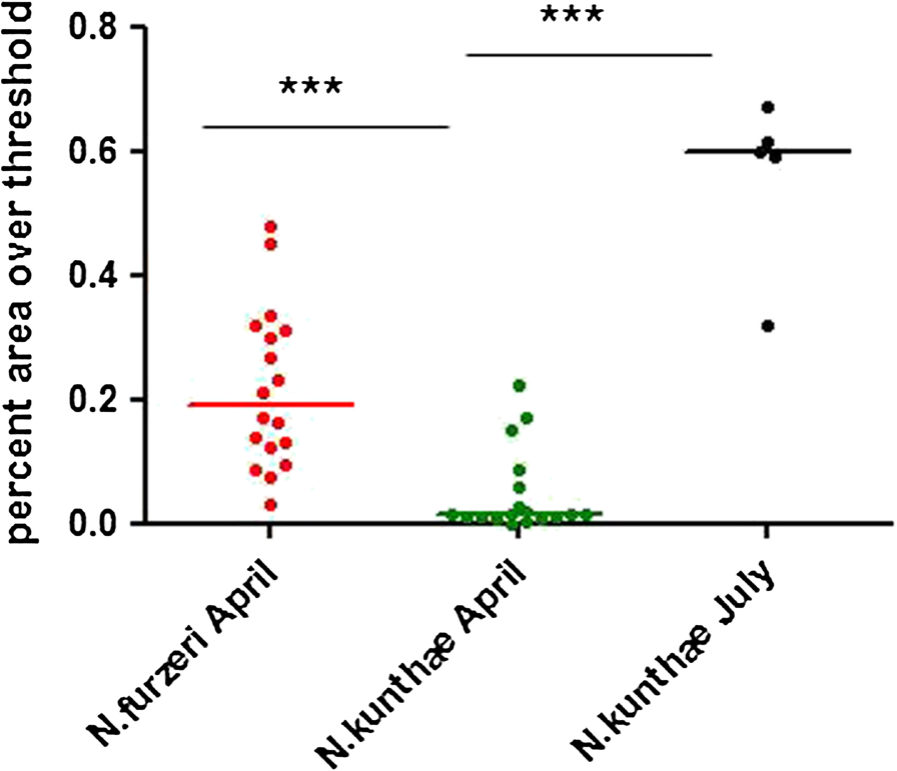
**Expression of lipofuscin in wild animals.** Lipofuscin measurements in the liver of wild individuals of *N. furzeri* and *N. kuhntae*. ***p = 0.001, Kruskall-Wallis non-parametric ANOVA.

To confirm that data on captive populations reflects natural processes, we collected wild individuals of *N*. *furzeri* and *N*. *kuhntae* in mid-April 2011. Since fish hatch synchronously around January ([23] and our current data) all fish had an estimated age of 15 weeks. We found a robust difference in lipofuscin load in liver between the populations (Figure 5, Mann-Whitney U-test, p < 0.001). We additionally analyzed *N*. *kuhntae* collected in July 2011 (estimated age of 24-25 weeks) and found more and much larger lipofuscin deposits in their liver than the April 2011 sample (Figure 5, Mann-Whitney U-test, p < 0.001). That age cohort of *N*. *furzeri* was already not present in the semi-arid region due to habitat desiccation.

### Genetic differentiation of *Nothobranchius* populations

The existence of parallel evolution of senescence in *Nothobranchius* may represent a paradigm for identification of loci under differential selection in short- and longer-lived species. This can be obtained by interspecific crosses and mapping of quantitative trait loci (QTL) or analysis of sequence variation. We therefore set to provide proof-of-principle for these two approaches.

A microsatellite-based linkage map was developed for *N*. *furzeri* in order to map *loci* controlling sex determination and color morphs in this species [39]. Species within *N*. *furzeri* and *N*. *kuhntae* lineage (further including *N*. *orthonotus* and *N*. *kadleci*), can be crossed in the laboratory and give rise to viable offspring ([48], N’goma & Cellerino unpublished data). To test whether genetic markers generated for *N*. *furzeri* can be used in the other *Nothobranchius* species from Southern Mozambique, we typed randomly-selected microsatellites in one individual each of *N*. *furzeri*, *N*. *kuhntae*, *N*. *rachovii* and *N*. *pienaari*.

Out of 96 markers, 83 (86%) and 81 (81%) provided amplification in *N*. *kuhntae* and *N*. *rachovii* /*N*. *pienaari* respectively. Out of 96 markers, 79 (82%) and 66 (68%), were polymorphic among the typed individuals and would have been informative in a prospective interspecific cross (Additional file 10: Table S10). Therefore, genetic markers which are currently being generated in *N*. *furzeri*[39,41] can be used for genetic studies in the other *Nothobranchius* species. To test for genome-wide nucleotide divergence between *N*. *furzeri* and *N*. *kuhntae*, we employed a genomic sequence sample of 6,357 *N*. *kuhntae* sequences (average length 791 bp, total length 5.03 Mb) generated previously [38]. These sequences were compared with a recently developed transcript catalogue of *N*. *furzeri* (A. Petzold *et al*., submitted) to identify protein-coding exons. There were 115 *N*. *kuhntae* genomic sequences (tBLASTx, p-value < e-20) which corresponded at least two exons (or parts thereof) in *N*. *furzeri* transcripts and could be aligned in the two species (total length of 44.7 kb). In these, we measured a mean nucleotide identity of 99%. The median frequency of estimated non-synonymous substitution per non-synonymous site (*Ka*) was 0.0032 and the median frequency of estimated synonymous substitution per synonymous site (*Ks*) was 0.0245 with *ω* = 0.13 (Additional file 11: Table S11) indicating on average strong purifying selection.

## Discussion and conclusion

In summary, we present evidence of parallel evolution of life span and senescence in annual *Nothobranchius* fishes. This evidence is based on interspecific comparison between closely related species inhabiting contrasting habitats, using two evolutionary lineages and replication of populations for each study species. In both lineages, the species from semi-arid habitat showed accelerated senescence compared to the species from a humid habitat. These data suggest that constraints to the maximum natural lifespan modulate the evolution of aging in these annual fish.

None of the wild-derived *N*. *furzeri* populations replicated the extremely short lifespan (3 months) observed in the laboratory strain GRZ [42] despite the fact that one population in our study (population MZZW 07/01) originates from just 30 km from the original sampling site of GRZ. The most straightforward explanation for these data is that the GRZ strain is highly inbred, probably due to the long captive history (40 years). Indeed, the GRZ strain is nearly completely homozygous [38,39]. However, we cannot exclude that a rare combination of natural alleles has been fixed in the GRZ strain that is known to have undergone at least one bottleneck where the entire captive population was constituted by a single breeding pair [49].

Ageing evolves as a trade-off in response to patterns of extrinsic mortality. Higher extrinsic mortality means that fewer individuals survive to reproduce at later ages, leading to erosion in selection to promote longevity and limit ageing effects. Prevailing theories [9,10,14] postulate that populations experiencing high extrinsic mortality evolve rapid ageing. Rapid ageing manifests itself by higher intrinsic mortality and more rapid deterioration of vital functions. Our data provide a strong quantitative example of modulation of aging by extrinsic mortality in vertebrates at an interspecific level. At intraspecific level, garter snake (*Thamnophis elegans*) populations from low- and high- extrinsic mortality environments were found to evolve long and short lifespans [50,51], while guppy populations from low- and high- extrinsic mortality environments provided ambiguous results [6,21]. The difference between the studies appears to be connected to the source of mortality. While the guppy survival may be strongly condition-dependent (predation pressure), a source of the garter snake mortality likely stems from environmental conditions (climatic factors, food abundance) and may be less prone to condition-dependence. This is also the case in our study system where the highest risk of mortality comes from habitat desiccation, impacting all individuals equally. Considering the source of mortality rather than only its strength appears to be crucial to understand the evolution of ageing at intra- and inter-specific levels [18,52].

Lipofuscin is known as the age pigment because it accumulates with age in many organisms from *C*. *elegans* to humans. Lipofuscin accumulation as a function of age is well studied in aquatic organisms [28,29,43,44,53-56] and *N*. *furzeri* is characterized by a rapid and massive accumulation of lipofuscin as a function of age [37]. We have demonstrated that lipofuscin accumulation in liver and brain is positively associated with mortality rates of our study populations. We further demonstrated that lipofuscin also accumulates in fish collected in the wild and is therefore a naturally occurring phenomenon rather than a consequence of particular diet in captivity. This makes the use of lipofuscin accumulation a suitable proxy of age-dependent deterioration not only in animals in captivity, but also in the wild.

Our inferences are based on a contrast in habitat duration between two regions. Our study design was initially based on indirect estimates of habitat duration between the two regions, such as annual rainfall totals (lower in semi-arid region), seasonal distribution of rainfall (more erratic in semi-arid region), evapotranspiration rates (higher in semi-arid region) and vegetation (plant communities known to be adapted to more arid conditions are present in semi-arid region) [37]. However, such data are not conclusive and other factors, such as local soil conditions or morphology of the pools (depth, size), may override those climatic factors. Here, we present data that confirmed strong differences in habitat duration between the two regions. Large dataset from field sites visits, collected over two years, clearly demonstrated that most *Nothobranchius* pools (67-71%) were dry in the semi-arid region in June or July, while only 25% of pools were dry in humid region. An additional relevant point is the synchronous hatch of *Nothobranchius* across the study region. Data on age of *Nothobranchius* fishes estimated from otoliths (ear structures that deposits daily increments) provided strong indication of synchronous hatching in the years 2008 and 2009 [23]. Here, we use datalogger records to document a synchronous start of the aquatic phase in both habitats, associated with enormous, cyclone-associated precipitation in 2012. A combination of these data demonstrated much shorter duration of pools (and fish therein contained) in the semi-arid region.

*Nothobranchius* are laboratory species and could be used to unravel the genetic architecture of these lifespan differences using two different approaches, for which we provide proof of principle. The first strategy is the use of intra- and inter-specific crossing between population/species with divergent phenotypes and subsequent analysis of quantitative trait loci (QTLs). This approach has led to characterization of the genetic architecture controlling natural phenotypic variation in several fish species [57-63]. Some QTLs controlling longevity were recently identified using a crossing panel of *N*. *furzeri* laboratory strains [41], but these crosses involved the GRZ strain and may not be representative of natural variations. The existence of a phenomenon of parallel evolution of senescence in *Nothobranchius* from Mozambique offers a powerful paradigm to identify conserved QTLs controlling longevity which may eventually lead to the identification of genes controlling natural variation in lifespan in vertebrates. The second approach is the use of genome-wide sequence comparisons of coding exons. If a specific gene in two lineages experiences a difference in selection pressure (either increased or decreased selection) a signature could be detected in the sequence, for example in the ratio of synonymous to non-synonymous substitutions (*Ka*/*Ks*). This method has already allowed the identification of gene under differential selection in long-living mammals [64]. A transcript catalogue of *N*. *furzeri* is now available (A. Petzold *et al*., submitted) and we measured here 99% sequence identity in the coding regions between *N*. *furzeri* and *N*. *kuhntae*. It will be straight-forward to use next generation sequencing techniques to identify transcripts with high *Ka*/*Ks* ratio and use them in subsequent anal*y*ses.

## Methods

### Habitat desiccation

In addition to clear pattern in precipitation differences between semi-arid and humid regions (Figure 1) and differences in evaporation-precipitation ratio [37], we used two complementary approaches to directly confirm that pools in humid region hold water for longer time that pools in semi-arid region.

First, we visited sites at the beginning of the dry season and recorded whether particular habitats have desiccated or not. Sites were visited between 7 and 11 July 2011 and between 25 May and 7 June 2012 in both regions (Table 1).

Second, waterproof dataloggers (Onset HOBO UA-001-08 and HOBO U22-001) were installed in pools across study regions to recover time of the pool desiccation. In 2011, 7 loggers were successfully read in semi-arid region and 2 loggers were read in humid region. In 2012, 12 loggers were successfully read in semi-arid region and 4 loggers were read in humid region (Additional file 12: Table S1). Dataloggers record water temperature every 90 or 180 minutes. In dry pools, extreme oscillations of the air temperature are observed. When the pool fills with water, there is an abrupt decrease in daily oscillations and this is clearly detectable from the records (Figure 2). Disappearance of water from the pond is marked by an increase in temperature fluctuation. This transition may be not as sharp as for filling (when logger is deposited in a wet mud), but given the large differences (in the order of months) across geographic regions, a potential inaccuracy is minor.

### Fish culture and survival assay

Some of the strains used for the experiments were collected by the authors and the generation is known (Table 2). Other strains were obtained via breeders specialized in killifish and in this case only the year of collection is known (Table 2). Eggs were maintained on wet peat moss at room temperature in sealed Petri dishes. When embryos had developed, eggs were hatched by flushing the peat with tap water at 16–18°C. Embryos were scooped up and transferred to a clean tank. Fry were fed with newly hatched *Artemia* nauplii for the first 2 weeks and then weaned with finely chopped *Chironomus* larvae. After four weeks (coincident with sexual maturity) fish were moved to 40-l tanks at a maximum density of 20 fishes per tank. Filtration was provided with air-driven sponge filters. The temperature was maintained at 25°C by climatizing the entire room. All assays were performed in the same fishroom on eggs hatched between June 2008 and November 2008. Light/dark cycles were maintained at 12:12.

Fish were fed twice a day with frozen *Chironomus* larvae at a quantity that they consumed in 30 min. *Chironomus* larvae were purchased from Poseidon Aquakultur (Ruppichterot, Germany). Twice a week the bottom of the tanks was siphoned and 50% of the water was exchanged with tempered tap water. Surviving fish were counted every week starting from the fifth week. Dead fish were not counted because they decay fast in water and may be eaten by their tank mates before they are noticed. To compute differences among treatments, we used commercially available GraphPad program. In particular, life tables containing censored data were generated by using as input either the week of death for fish that died naturally or the week of sacrifice for censored data. Differences in survivorships were evaluated by Log-rank and Mantel-Cox tests without correction for multiple testing.

### Histology and lipofuscin quantification

Fishes raised in captivity were euthanized with MS-222 and cooled on crushed ice for 5 min before dissection. Target tissues were dissected and fixed by immersion in 4% paraformaldehyde/0.1 M phosphate buffer (pH 7.4). Fish collected in the wild were euthanized with clove oil, a slit was cut in the abdomen to allow fixative penetration and the entire body was fixed in Baker’s solution. Fish tissues were embedded in Paraplast and sections of 5 mm in thickness were cut.

Intracellular accumulation of lipofuscin during aging was detected in brain and liver tissues from young and old fishes of all different populations of the two groups of closely related species (from humid and semi-arid habitats). Lipofuscin is normally detected as light blue autofluorescent granules under UV excitation. For quantification, images were acquired using a Leica confocal microscope (in Pisa, wild animals) or a Zeiss LSM (in Jena, captive animals) at an excitation wavelength of 488 nm, with fixed confocal parameters (pinhole, photo-multiplier, laser intensity, etc.). Digital images were manually edited to remove autofluorescent erythrocytes as described in [37]. Image analysis was performed in Image J (http://rsbweb.nih.gov/ij/) by setting a fixed value of fluorescence as threshold identical for all pictures and then by quantification of the percentage of area over threshold. Statistical analysis was performed in GraphPad.

### Genetic differentiation of *Nothobranchius* populations

Ninety-six microsatellites were genotyped: 56 FLI, and 40 SU. All microsatellite markers were previously identified at Fritz Lipman Institute, FLI [38] and Stanford University, SU [39]. PCR reactions were performed in 13 μl final volumes in 96-well plates, each with 1x PCR buffer, 1.5 mM MgCl2, 10 mM each dNTP mix (Fermentas), 0.2 μM M13(-21) adaptor (Schuelke 2000), 0.06 μM FAM-labeled M13(-21) forward primer, 0.2 μM reverse primer, 1.25 units Taq DNA Polymerase (Qiagen) and 100-150 ng of genomic DNA. PCR conditions were 94°C, 5 min initial denaturation followed by 35 cycles of 94°C, 30 s, denaturation; 56°C 30 s, annealing; 72°C, 60 s, extension; and a final extension step of 1 hour at 72°C. Amplicons were denatured by incubation in 10 μ Hi-Di Formamide (Roth) and 0.5 μl GeneScan-500 ROX Size Standard (Applied Biosystems) at 95°C for 5 min and electrophoresed on an ABI 3730 capillary sequencer. GeneScan software (Applied Biosystems) was used to visualize and score chromatographs.

## Competing interests

The authors declare that they have no competing interests.

## Authors’ contributions

ETT designed the study, performed histological analysis and lifespan recordings, AD performed lifespan recordings, EN performed microsatellite analysis, BW provided biological material, MP and RB performed field studies, KR and AP performed sequence analysis, MR provided biological material, performed field studies and wrote the paper, AC designed and supervised the study, analysed data and wrote the paper. All authors read and approved the final manuscript.

## Supplementary Material

Additional file 1: Table S2 Life span and sample size of the captive populations of the *N*. *furzeri*/*N*. *kuhntae* clade used for the study. Click here for file

Additional file 2: Table S3 Pairwise Log-rank statistics of survivorship for all populations of the *N*. *furzeri*/*N*. *kunthae* clade in the study. FUR = *N*. *furzeri*, KUN = *N*. *Kuhntae*. Click here for file

Additional file 3: Figure S1 Age-dependent mortality in *N*. *furziri* and *N*. *kuhntae*. The lines report smoothed mortality curves for the two species. Each point represents the average of four consecutive weeks. In order to allow exponential fit, zero values were substituted with 0.001. Click here for file

Additional file 4:Table S4 Life span and sample size of the captive populations of the *N*. *pienaari*/*N*. *rachovii* clade used for the study. Click here for file

Additional file 5: Table S5 Pairwise Log-rank statistics of survivorship for all populations of the *N*. *pienaari*/*N*. *rachovii* clade used for the study. PIE = *N*. *pienaari*, RAC = *N*. *Rachovii*. Click here for file

Additional file 6: Table S6 Kruskall-Wallis ANOVA of lipofuscin accumulation in the liver, pair-wise comparisons for all the populations of the *N*. *furzeri*/*N*. *kunthae* clade in the study. FUR = *N*. *furzeri*, KUN = *N*. *Kuhntae*. Click here for file

Additional file 7: Table S7 Kruskall-Wallis ANOVA of lipofuscin accumulation in the liver, pair-wise comparisons for all the populations of the *N*. *pienaari*/*N*. *rachovii* clade used for the study. PIE = *N*. *pienaari*, RAC = *N*. *rachovii*. Click here for file

Additional file 8: Table S8 Kruskall-Wallis ANOVA of lipofuscin accumulation in the brain, pair-wise comparisons for all the populations of the *N*. *furzeri*/*N*. *kunthae* clade in the study. FUR = *N*. *furzeri*, KUN = *N*. *kuhntae*. Click here for file

Additional file 9: Table S9 Kruskall-Wallis ANOVA of lipofuscin accumulation in the brain, pair-wise comparisons for all the populations of the *N*. *pienaari*/*N*. *rachovii* clade used for the study. PIE = *N*. *pienaari*, RAC = *N*. *rachovii*. Click here for file

Additional file 10: Table S10 Microsatellite polymorphisms in the four studied species - Microsatellites were amplified using PCR on genomic DNA and microsatellites ID are described in Valenzano et al. (2009). For each of the two alleles, the length of the amplified fragment is reported. For homozygous loci, both these values are identical. One individual for each species was analyzed, n.a. indicates no amplification. Click here for file

Additional file 11: Table S11 Sequence divergence between *N*. *furzeri* and *N*. *kuhntae* measured at 115 loci - Column 1 reports the gene name, Column 2 reports the length of the coding sequence in *N*. *furzeri*, column 3 reports the length of the alignment between the two ortholog sequences, column 4 reports the percentage of the *N*. *furzeri* coding sequence covered by the alignment, column 5 reports the ID of the *N*. *furzeri* transcript, column 6 reports the ID of the *N*. *kunthae* genomic contig, column 7 reports the number of sites of possible non-synonimous substitution, column 8 reports the frequency of non-synonimous substituions per site, column 9 reports the number of sites of possible synonimous substitution (dN), column 10 reports the frequency of synonimous substituions per site (dS), column 10 reports the dN/dS ratio, column 11 reports the percentage of aminoacidic sequence identity in the alignment, column 12 reports the percentage of nucleotide sequence identity in the alignment, column 12 reports the number of nonsynonimous sequence variations and column 13 the number of synonymous sequence variations. The last two rows report the median of all columns and the total for columns 2, 3, 6, 8, 13 and 14. Click here for file

Additional file 12: Table S1 The number of dataloggers which were still submerged (water) and loggers exposed on dry bottom (dry) recovered at each region. Click here for file
