# Supplementary material for: Parallel evolution of senescence in annual fishes in response to extrinsic mortality
Source: BMC Evol Biol. 2013 Apr 3;13:77. doi: 10.1186/1471-2148-13-77 (PMC3623659; doi:10.1186/1471-2148-13-77)
Supplement: Additional file 1: Table S2 — Life span and sample size of the captive populations of the N. furzeri/N. kuhntae clade used for the study. [file 1471-2148-13-77-S1.docx]

**Table S2** Life span and sample size of the captive populations of the *N. furzeri*/*N. kuhntae* clade used for the study.

| **Species** | **strain** |  | **n** | **median** | **10% surv.** |
| --- | --- | --- | --- | --- | --- |
| ***N. furzeri*** | **MZZW 07/01** |  | 124 | 17.5 | 33 |
| ***N. furzeri*** | **MZM 04/10** |  | 113 | 29 | 40 |
| ***N. furzeri*** | **MZCS 08/122** |  | 33 | 28 | 40 |
| ***N. kuhntae*** | **MT 03/04** |  | 23 | n.d. | n.d. |
| ***N. kuhntae*** | **Aquarium Strain (AS)** |  | 25 | 42 | 57 |
| ***N. kuhntae*** | **MOZ 04/07** |  | 24 | 49 | 50 |

*=experiment interrupted when animals at 33 weeks of age due to disease outbreak in the tank.
