# Supplementary material for: Parallel evolution of senescence in annual fishes in response to extrinsic mortality
Source: BMC Evol Biol. 2013 Apr 3;13:77. doi: 10.1186/1471-2148-13-77 (PMC3623659; doi:10.1186/1471-2148-13-77)
Supplement: Additional file 2: Table S3 — Pairwise Log-rank statistics of survivorship for all populations of the N. furzeri/N. kunthae clade in the study. FUR = N. furzeri, KUN = N. Kuhntae. [file 1471-2148-13-77-S2.docx]

**Table S3** Pairwise Log-rank statistics of survivorship for all populations of the *N. furzeri*/*N. kunthae* clade in the study. FUR = *N. furzeri*, KUN = *N. Kuhntae*

| **Species** | **Strain** | **FUR**  **MZZW** | **FUR**  **MZM** | **FUR**  **MZCS** | **KUN**  **MT** | **KUN**  **AS** | **KUN**  **MOZ** |
| --- | --- | --- | --- | --- | --- | --- | --- |
| ***N. furzeri*** | **MZZW 07/01** | - | p<0.0001 | p<0.0001 | p<0.0001 | p<0.0001 | p<0.0001 |
| ***N. furzeri*** | **MZM 04/10** |  | - | n.s. | p=0.08 | p<0.001 | p<0.0001 |
| ***N. furzeri*** | **MZCS 08/122** |  |  | - | p=0.08 | p=0.0006 | p<0.0001 |
| ***N. kuhntae*** | **MT 03/04** |  |  |  | - | n.s. | n.s. |
| ***N. kuhntae*** | **AS** |  |  |  |  | - | n.s. |
| ***N. kuhntae*** | **MOZ 04/07** |  |  |  |  |  | - |

.
