# Supplementary figures and images for: Parallel evolution of senescence in annual fishes in response to extrinsic mortality
Source: BMC Evol Biol. 2013 Apr 3;13:77. doi: 10.1186/1471-2148-13-77 (PMC3623659; doi:10.1186/1471-2148-13-77)

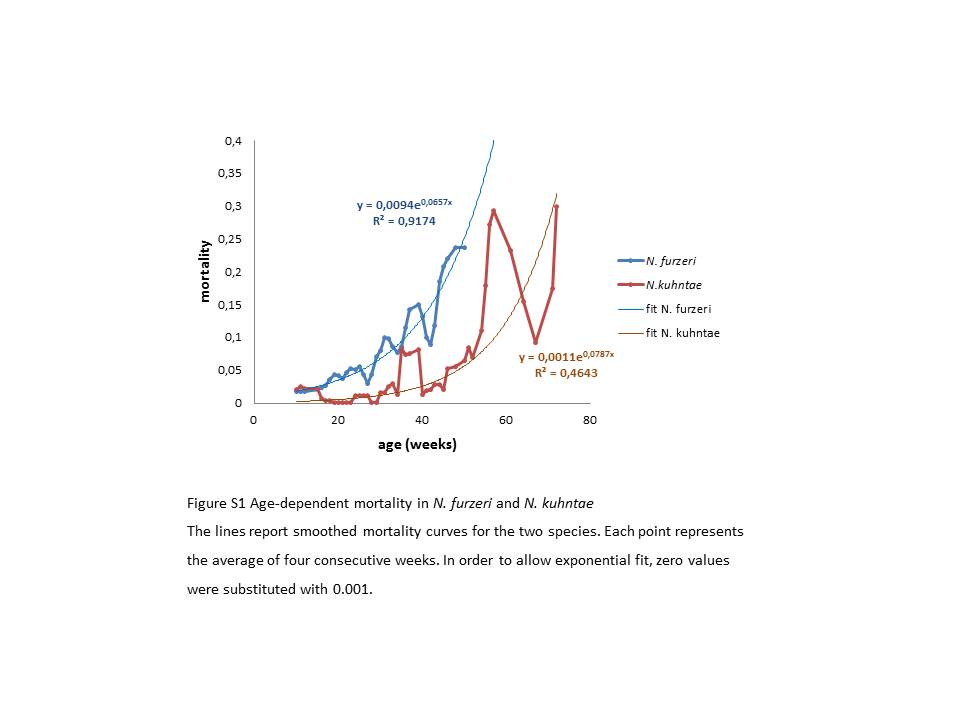

Supplement: Additional file 3: Figure S1 — Age-dependent mortality in N. furziri and N. kuhntae. The lines report smoothed mortality curves for the two species. Each point represents the average of four consecutive weeks. In order to allow exponential fit, zero values were substituted with 0.001. [file 1471-2148-13-77-S3.jpeg]
