# Supplementary material for: Parallel evolution of senescence in annual fishes in response to extrinsic mortality
Source: BMC Evol Biol. 2013 Apr 3;13:77. doi: 10.1186/1471-2148-13-77 (PMC3623659; doi:10.1186/1471-2148-13-77)
Supplement: Additional file 4:Table S4 — Life span and sample size of the captive populations of the N. pienaari/N. rachovii clade used for the study. [file 1471-2148-13-77-S4.docx]

**Table S4** Life span and sample size of the captive populations of the *N. pienaari*/*N. rachovii* clade used for the study

| **Species** | **strain** | **n** | **median** | **10%surv.** |
| --- | --- | --- | --- | --- |
| ***N. pienaari*** | **MOZ 99/3** | 61 | 33 | 43 |
| ***N. pienaari*** | **MOZ 99/9** | 31 | 41 | 55 |
| ***N. rachovii*** | **MT 03/01** | 43 | 40 | 63 |
| ***N. rachovii*** | **Beira 98** | 34 | 55 | 65 |
