# Supplementary material for: Parallel evolution of senescence in annual fishes in response to extrinsic mortality
Source: BMC Evol Biol. 2013 Apr 3;13:77. doi: 10.1186/1471-2148-13-77 (PMC3623659; doi:10.1186/1471-2148-13-77)
Supplement: Additional file 5: Table S5 — Pairwise Log-rank statistics of survivorship for all populations of the N. pienaari/N. rachovii clade used for the study. PIE = N. pienaari, RAC = N. Rachovii. [file 1471-2148-13-77-S5.docx]

**Table S5** Pairwise Log-rank statistics of survivorship for all populations of the *N. pienaari*/*N. rachovii* clade used for the study. PIE = *N. pienaari*, RAC = *N. Rachovii*

| **Species** | **Strains** | **PIE**  **MOZ 99/3** | **PIE**  **MOZ 99/9** | **RAC**  **MT 03/01** | **RAC**  **Beira 98** | ***N.rachovii* pooled** |
| --- | --- | --- | --- | --- | --- | --- |
| ***N. pienaari*** | **MOZ 99/3** | - | p=0.0005 | p<0.0001 | p<0.0001 | p<0.0001 |
| ***N. pienaari*** | **MOZ 99/9** |  | - | n.s | p=0.004 | p=0.019 |
| ***N. rachovii*** | **MT 03/01** |  |  | - | n.s | - |
| ***N. rachovii*** | **Beira 98** |  |  |  | - | - |

*.*
