# Supplementary material for: Parallel evolution of senescence in annual fishes in response to extrinsic mortality
Source: BMC Evol Biol. 2013 Apr 3;13:77. doi: 10.1186/1471-2148-13-77 (PMC3623659; doi:10.1186/1471-2148-13-77)
Supplement: Additional file 7: Table S7 — Kruskall-Wallis ANOVA of lipofuscin accumulation in the liver, pair-wise comparisons for all the populations of the N. pienaari/N. rachovii clade used for the study. PIE = N. pienaari, RAC = N. rachovii. [file 1471-2148-13-77-S7.docx]

**Table S7**  Kruskall-Wallis ANOVA of lipofuscin accumulation in the liver, pair-wise comparisons for all the populations of the *N. pienaari*/*N. rachovii* clade used for the study. PIE = *N. pienaari*, RAC = *N. rachovii.*

|  | **PIE**  **MOZ 99/1** | **PIE**  **MOZ 99/3** | **RAC**  **Beira 98** | **RAC**  **MT 03/01** |
| --- | --- | --- | --- | --- |
| **PIE MOZ 99/1** | - | n.s. | * | *** |
| **PIE MOZ 99/3** |  | - | *** | *** |
| **RAC Beira 98** |  |  | - | n.s. |
| **RAC MT 03/01** |  |  |  | - |
