# Supplementary material for: Parallel evolution of senescence in annual fishes in response to extrinsic mortality
Source: BMC Evol Biol. 2013 Apr 3;13:77. doi: 10.1186/1471-2148-13-77 (PMC3623659; doi:10.1186/1471-2148-13-77)
Supplement: Additional file 8: Table S8 — Kruskall-Wallis ANOVA of lipofuscin accumulation in the brain, pair-wise comparisons for all the populations of the N. furzeri/N. kunthae clade in the study. FUR = N. furzeri, KUN = N. kuhntae. [file 1471-2148-13-77-S8.docx]

**Table S8** Kruskall-Wallis ANOVA of lipofuscin accumulation in the brain, pair-wise comparisons for all the populations of the *N. furzeri*/*N. kunthae* clade in the study. FUR = *N. furzeri*, KUN = *N. kuhntae*.

|  | **KUN**  **MT 03/02** | **KUN**  **MOZ 04/07** | **FUR**  **MOZ 04/10** | **FUR**  **MZZW 07/01** |
| --- | --- | --- | --- | --- |
| **KUN MT 03/02** | - | n.s. | *** | *** |
| **KUN MOZ 04/07** |  | - | *** | *** |
| **FUR MOZ 04/10** |  |  | - | * |
| **FUR MZZW 07/01** |  |  |  | - |
