# Supplementary material for: Parallel evolution of senescence in annual fishes in response to extrinsic mortality
Source: BMC Evol Biol. 2013 Apr 3;13:77. doi: 10.1186/1471-2148-13-77 (PMC3623659; doi:10.1186/1471-2148-13-77)
Supplement: Additional file 10: Table S10 — Microsatellite polymorphisms in the four studied species - Microsatellites were amplified using PCR on genomic DNA and microsatellites ID are described in Valenzano et al. (2009). For each of the two alleles, the length of the amplified fragment is reported. For homozygous loci, both these values are identical. One individual for each species was analyzed, n.a. indicates no amplification. [file 1471-2148-13-77-S10.pdf]

Table S10 Microsatellite polymorphisms in the four studied species - Microsatellites were amplified using PCR on genomic DNA and microsatellites ID are described in Valenzano et al. (2009). For each of the two alleles, the length of the amplified fragment is reported. For homozygous loci, both these values are identical. One individual for each species was analyzed, n.a. indicates no amplification.

microsatellite ID[1]		amplicon size			
		N. furzeri GRZ	*N. kuhntae*	*N.rachovii*	*N.pienaari*
FLI001	allele1	189	160	162	161
	allele2	189	162	162	176
FLI006	allele1	298	303	289	359
	allele2	298	303	293	359
FLI008	allele1	214	n.a.	n.a.	n.a.
	allele2	214	n.a.	n.a.	n.a.
FLI009	allele1	192	171	233	251
	allele2	192	185	233	251
FLI010	allele1	211	189	205	187
	allele2	211	215	269	269
FLI012	allele1	203	n.a.	n.a.	n.a.
	allele2	203	n.a.	n.a.	n.a.
FLI013	allele1	240	418	388	420
	allele2	240	418	418	420
FLI015	allele1	245	275	313	286
	allele2	245	357	357	296
FLI016	allele1	244	257	249	235
	allele2	244	257	257	235
FLI017	allele1	263	359	289	303
	allele2	263	359	293	303
FLI019	allele1	259	198	210	190
	allele2	259	198	226	190
FLI020	allele1	248	n.a.	n.a.	n.a.
	allele2	248	n.a.	n.a.	n.a.
FLI021	allele1	292	287	297	271
	allele2	292	287	309	315
FLI024	allele1	250	248	292	316
	allele2	250	248	298	316
FLI026	allele1	187	n.a.	n.a.	n.a.
	allele2	187	n.a.	n.a.	n.a.
FLI027	allele1	237	188	202	314
	allele2	237	220	202	314
FLI028	allele1	244	288	n.a.	n.a.
	allele2	244	296	n.a.	n.a.
FLI030	allele1	188	163	167	185
	allele2	188	163	173	197
FLI033	allele1	232	242	210	218
	allele2	232	246	210	218
FLI036	allele1	346	330	384	378
	allele2	346	330	396	378
FLI041	allele1	205	251	n.a.	245
	allele2	205	251	n.a.	245
FLI045	allele1	388	370	378	368
	allele2	388	370	388	368
FLI046	allele1	563	n.a.	n.a.	n.a.
	allele2	563	n.a.	n.a.	n.a.
FLI049	allele1	341	295	285	293
	allele2	341	295	289	295
FLI050	allele1	395	n.a.	n.a.	n.a.
	allele2	395	n.a.	n.a.	n.a.
FLI052	allele1	348	436	405	446
	allele2	348	436	408	462
FLI053	allele1	288	362	n.a.	382

	allele2	288	362	n.a.	382
FLI055	allele1	227	n.a.	n.a.	n.a.
	allele2	227	n.a.	n.a.	n.a.
FLI060	allele1	434	415	363	383
	allele2	434	415	381	393
FLI062	allele1	414	391	389	363
	allele2	414	391	389	363
FLI063	allele1	432	n.a.	n.a.	n.a.
	allele2	432	n.a.	n.a.	n.a.
FLI067	allele1	358	398	432	398
	allele2	358	398	432	398
FLI069	allele1	378	485	355	425
	allele2	378	485	411	475
FLI070	allele1	318	359	359	365
	allele2	318	359	359	365
FLI071	allele1	503	n.a.	n.a.	n.a.
	allele2	503	n.a.	n.a.	n.a.
FLI073	allele1	301	309	333	291
	allele2	301	309	341	291
FLI076	allele1	469	n.a.	n.a.	n.a.
	allele2	469	n.a.	n.a.	n.a.
FLI078	allele1	283	247	252	255
	allele2	283	250	252	262
FLI080	allele1	340	299	229	283
	allele2	340	407	229	283
FLI090	allele1	300	296	316	334
	allele2	300	316	316	334
FLI093	allele1	409	417	413	413
	allele2	409	417	423	417
FLI095	allele1	491	418	402	440
	allele2	491	476	402	528
FLI096	allele1	302	329	275	313
	allele2	302	329	275	313
FLI097	allele1	401	509	383	395
	allele2	401	513	383	425
FLI102	allele1	461	479	453	491
	allele2	461	491	453	491
FLI104	allele1	264	307	297	299
	allele2	264	347	357	299
FLI107	allele1	460	438	438	442
	allele2	460	442	460	442
FLI108	allele1	280	240	336	300
	allele2	280	240	336	300
FLI110	allele1	210	200	188	198
	allele2	210	230	204	228
FLI113	allele1	514	504	300	300
	allele2	514	504	300	300
FLI121	allele1	406	416	410	416
	allele2	406	416	410	416
FLI123	allele1	255	236	238	246
	allele2	255	242	238	246
FLI124	allele1	540	547	577	577
	allele2	540	555	577	577
FLI128	allele1	417	378	382	448
	allele2	417	378	382	448
FLI132	allele1	433	n.a.	n.a.	n.a.

	allele2	433	n.a.	n.a.	n.a.
FLI134	allele1	303	211	267	211
	allele2	303	211	267	295
SU001	allele1	207	211	211	257
	allele2	207	211	233	257
SU002	allele1	250	186	164	240
	allele2	250	186	250	250
SU007	allele1	181	219	205	205
	allele2	191	255	225	219
SU008	allele1	253	243	169	163
	allele2	253	243	199	243
SU010	allele1	199	167	243	215
	allele2	251	195	243	243
SU012	allele1	211	199	173	189
	allele2	211	209	221	217
SU014	allele1	223	228	218	228
	allele2	223	228	218	236
SU017	allele1	263	168	168	298
	allele2	263	168	168	318
SU027	allele1	226	183	161	163
	allele2	226	231	163	211
SU031	allele1	123	211	165	171
	allele2	123	211	179	203
SU032	allele1	256	268	248	276
	allele2	256	268	254	294
SU033	allele1	230	163	209	159
	allele2	230	209	209	187
SU034	allele1	318	222	248	214
	allele2	318	240	252	214
SU037	allele1	331	216	222	234
	allele2	331	216	222	234
SU038	allele1	118	219	117	219
	allele2	118	219	117	225
SU039	allele1	203	228	178	184
	allele2	203	228	178	184
SU040	allele1	171	117	117	169
	allele2	171	117	117	169
SU046	allele1	210	199	221	181
	allele2	210	219	221	209
SU054	allele1	156	138	122	126
	allele2	156	152	230	126
SU055	allele1	192	203	241	185
	allele2	192	209	241	197
SU056	allele1	188	196	160	164
	allele2	188	204	160	164
SU058	allele1	209	176	223	n.a.
	allele2	209	176	267	n.a.
SU059	allele1	222	139	157	153
	allele2	222	199	223	197
SU061	allele1	242	317	315	305
	allele2	242	333	327	317
SU062	allele1	250	279	296	307
	allele2	250	279	296	307
SU064	allele1	237	248	224	190
	allele2	237	248	232	196
SU065	allele1	181	203	205	205

	allele2	181	205	205	211
SU067	allele1	207	191	205	183
	allele2	207	191	205	205
SU069	allele1	252	251	248	251
	allele2	252	251	248	251
SU071	allele1	258	361	n.a.	373
	allele2	258	361	n.a.	373
SU072	allele1	209	226	368	206
	allele2	209	226	368	230
SU073	allele1	273	n.a.	264	250
	allele2	273	n.a.	264	250
SU075	allele1	388	410	410	310
	allele2	388	410	410	316
SU077	allele1	194	222	222	276
	allele2	194	222	290	276
SU078	allele1	190	186	180	178
	allele2	190	188	180	178
SU079	allele1	306	328	298	280
	allele2	306	332	298	280
SU080	allele1	179	n.a.	n.a.	n.a.
	allele2	179	n.a.	n.a.	n.a.
SU081	allele1	191	186	180	178
	allele2	191	188	180	178
SU082	allele1	263	218	236	218
	allele2	263	276	236	266
SU083	allele1	222	235	223	205
	allele2	222	235	223	205
