# Supplementary material for: Parallel evolution of senescence in annual fishes in response to extrinsic mortality
Source: BMC Evol Biol. 2013 Apr 3;13:77. doi: 10.1186/1471-2148-13-77 (PMC3623659; doi:10.1186/1471-2148-13-77)
Supplement: Additional file 11: Table S11 — Sequence divergence between N. furzeri and N. kuhntae measured at 115 loci - Column 1 reports the gene name, Column 2 reports the length of the coding sequence in N. furzeri, column 3 reports the length of the alignment between the two ortholog sequences, column 4 reports the percentage of the N. furzeri coding sequence covered by the alignment, column 5 reports the ID of the N. furzeri transcript, column 6 reports the ID of the N. kunthae genomic contig, column 7 reports the number of sites of possible non-synonimous substitution, column 8 reports the frequency of non-synonimous substituions per site, column 9 reports the number of sites of possible synonimous substitution (dN), column 10 reports the frequency of synonimous substituions per site (dS), column 10 reports the dN/dS ratio, column 11 reports the percentage of aminoacidic sequence identity in the alignment, column 12 reports the percentage of nucleotide sequence identity in the alignment, column 12 reports the number of nonsynonimous sequence variations and column 13 the number of synonymous sequence variations. The last two rows report the median of all columns and the total for columns 2, 3, 6, 8, 13 and 14. [file 1471-2148-13-77-S11.pdf]

Table S11 Sequence divergence between *N. furzeri* and *N. kuhntae* measured at 115 loci - Column 1 reports the gene name, Column 2 reports the length of the coding sequence in *N. furzeri*, column 3 reports the length of the alignment between the two ortholog sequences, column 4 reports the percentage of the *N. furzeri* coding sequence covered by the alignment, column 5 reports the ID of the *N. furzeri* transcript, column 6 reports the ID of the *N. kunthae* genomic contig, column 7 reports the number of sites of possible non-synonimous substitution, column 8 reports the frequency of non-synonimous substituions per site, column 9 reports the number of sites of possible synonimous substitution (dN), column 10 reports the frequency of synonimous substituions per site (dS), column 10 reports the dN/dS ratio, column 11 reports the percentage of aminoacidic sequence identity in the alignment, column 12 reports the percentage of nucleotide sequence identity in the alignment, column 12 reports the number of non-synonimous sequence variations and column 13 the number of synonymous sequence variations. The last two rows report the median of all columns and the total for columns 2, 3, 6, 8, 13 and 14.

Gene	length [bp] cds in Nofu	length nt-ali seq1 to seq2	[%]	GRZ transcript ID: Nofu_GRZ_cDNA_3_	Noku genom contig	N = avg no. non-syn sites	dN (*K*a)	S = avg no. syn sites	dS (*Ks*)	dN/dS	prot_ID [%]	cDNA_ID [%]	# non-syn var	# syn var
ADAMTS12	4668	316	6.8	1250	Nku-G-d-27c01.f1	211.9	0.0000	103.1	0.0102	0.00100	100	99.68		1
KIAA1614	2928	243	8.3	4622	Nku-G-d-06g10.f1	168.8	0.0001	74.2	0.0557	0.00100	100	98.35	0	4
IKBKB	2214	548	24.8	176555	Nku-G-d-05c09.f1	437.4	0.0000	108.6	0.0186	0.00100	100	99.63	0	2
LLGL2	3012	212	7.0	160453	Nku-G-d-26e03.r1	156.6	0.0000	53.4	0.0392	0.00100	100	99.05	0	2
SPTBN2	6882	491	7.1	207523	Nku-G-d-22h11.r1	325.1	0.0000	163.9	0.0058	0.00100	100	99.8	0	1
ANKHA	1479	395	26.7	10472	Nku-G-d-04g10.f1	268.2	0.0000	124.8	0.0420	0.00100	100	98.73	0	5
RBMX2	846	254	30.0	195428	Nku-G-d-49d02.f1	197.6	0.0000	54.4	0.0182	0.00100	100	99.6	0	1
TXNL1	867	350	40.4	34863	Nku-G-d-01h03.f1	265.5	0.0000	82.5	0.0375	0.00100	100	99.14	0	3
DDX47 (1 OF 2)	1425	262	18.4	34410	Nku-G-d-16h04.r1	181.7	0.0000	79.3	0.0379	0.00100	100	98.85	0	3
RPRD2 (1 OF 2)	2139	420	19.6	182952	Nku-G-d-57d12.f1	272.5	0.0000	147.5	0.0147	0.00100	100	99.52	0	2
PSMC6	1182	202	17.1	7763	Nku-G-d-37b07.f1	151.0	0.0000	47.0	0.0226	0.00100	98.51	98.51	0	1
KDRL	3900	252	6.5	159935	Nku-G-d-18f02.r1	194.4	0.0001	57.6	0.0569	0.00100	100	98.81	0	3
CHD4 (2 OF 2)	5871	393	6.7	159809	Nku-G-d-11c07.f1	296.5	0.0000	96.5	0.0332	0.00100	100	99.24	0	3
DLG5	6144	572	9.3	207672	Nku-G-d-62c12.f1	396.2	0.0000	173.8	0.0117	0.00100	100	99.65	0	2
AGPS	1920	163	8.5	7906	Nku-G-d-56a07.r1	106.0	0.0000	56.0	0.0388	0.00100	100	98.77	0	2
EVPL (1 OF 2)	11169	302	2.7	179063	Nku-G-d-59b06.r1	237.1	0.0000	62.9	0.0165	0.00100	100	99.67	0	1
SI:CH211-66G24	3321	248	7.5	1798	Nku-G-d-46g09.f1	193.5	0.0001	52.5	0.0598	0.00100	100	98.78	0	3
TARSL2	2430	190	7.8	10756	Nku-G-d-01e09.r1	134.9	0.0000	54.1	0.0185	0.00100	100	99.47	0	1
MAST1 (1 OF 2)	7035	241	3.4	5653	Nku-G-d-01b02.r1	174.2	0.0000	65.8	0.0000	0.00100	100	100	0	0
MYO16	5880	202	3.4	175664	Nku-G-d-19a04.f1	128.4	0.0000	72.6	0.0133	0.00100	100	99.5	0	1
XPR1 (2 OF 2)	2073	306	14.8	1498	Nku-G-d-35h05.f1	250.0	0.0000	56.0	0.0375	0.00100	100	99.35	0	2
BMP1A	2010	345	17.2	4584	Nku-G-d-18g05.r1	280.4	0.0000	64.6	0.0480	0.00100	100	99.13	0	3
CNOT2 (2 OF 2)	1608	254	15.8	9641	Nku-G-d-26d05.f1	167.0	0.0000	85.0	0.0120	0.00100	100	99.6	0	1
CYFIP2	3720	353	9.5	4139	Nku-G-d-14c12.f1	248.1	0.0000	102.9	0.0102	0.00100	100	99.72	0	1
FPGS	1716	187	10.9	208283	Nku-G-d-47c10.f1	140.2	0.0000	45.8	0.0209	0.00100	100	99.46	0	1
TNFAIP3	2538	297	11.7	0185099-cds	Nku-G-d-15b04.r1-cds	201.6	0.0000	92.4	0.0342	0.00100	100	98.98	0	3
CDK20	2043	346	16.9	161411	Nku-G-d-01g08.r1	274.8	0.0000	70.2	0.0147	0.00100	100	99.71	0	1
GLT25D2	1857	338	18.2	193756	Nku-G-d-11e11.r1	221.1	0.0000	114.9	0.0179	0.00100	100	99.4	0	2
TFR1A	2271	402	17.7	4579	Nku-G-d-23c02.f1	321.4	0.0000	80.6	0.0399	0.00100	100	99.25	0	3
HELLS	2565	465	18.1	8615	Nku-G-d-01a10.r1	379.4	0.0001	85.6	0.0607	0.00100	100	98.92	0	5
APC	7590	1,142	15.0	175577	Nku-G-d-02d08.f1	874.0	0.0000	266.0	0.0302	0.00100	100	99.3	0	8
UFSP2	1383	458	33.1	193578	Nku-G-d-12a05.r1	347.1	0.0000	108.9	0.0194	0.00100	100	99.56	0	2
DIRAS1 (2 OF 2)	624	302	48.4	184264	Nku-G-d-31h06.r1	253.3	0.0000	46.7	0.0468	0.00100	100	99.33	0	2
GRID2IP (1 OF 2)	1959	255	13.0	706	Nku-G-d-21a04.r1	205.7	0.0000	49.3	0.0209	0.00100	100	99.61	0	1
ZNF365	1326	523	39.4	193079	Nku-G-d-25e02.f1	419.1	0.0000	102.9	0.0410	0.00100	100	99.23	0	4
LIMD2	342	291	85.1	53724	Nku-G-d-60b11.r1	206.3	0.0000	84.7	0.0115	0.00100	100	99.66	0	1
BTF3L4	480	316	65.8	16015	Nku-G-d-59f03.f1	221.9	0.0000	93.1	0.0104	0.00100	100	99.68	0	1
ZMYND11	1605	354	22.1	19182	Nku-G-d-62d01.f1	285.6	0.0035	68.4	0.0645	0.05363	99.15	98.59	1	4
FEN1	1140	381	33.4	209082	Nku-G-d-45g04.f1	271.4	0.0037	109.6	0.0585	0.06340	99.21	98.16	1	6
TMC6 (1 OF 2)	1665	467	28.0	193204	Nku-G-d-02f05.r1	357.3	0.0028	107.7	0.0393	0.07215	99.35	98.92	1	4
IRX6A	1392	661	47.5	177264	Nku-G-d-15d05.r1	480.9	0.0021	179.1	0.0286	0.07418	99.55	99.09	1	5
EFR3A	2460	282	11.5	192894	Nku-G-d-24b05.r1	223.3	0.0094	52.7	0.1245	0.07533	96.77	96.77	2	7
ARHGEF17	4413	355	8.0	73	Nku-G-d-07g12.r1	264.7	0.0038	89.3	0.0472	0.08115	99.15	98.59	1	4
ZNF385C	1437	567	39.5	178604	Nku-G-d-33f09.r1	396.1	0.0026	170.9	0.0305	0.08394	99.47	98.94	1	5
IGFN1	10053	295	2.9	1	Nku-G-d-36h05.f1	211.8	0.0048	82.2	0.0516	0.09382	98.98	98.3	1	4

Gene	length [bp] cds in Nofu	length nt-ali seq1 to seq2	[%]	GRZ transcript ID: Nofu_GRZ_cDNA_3_	Noku genom contig	N = avg no. non-syn sites	dN (*K*a)	S = avg no. syn sites	dS (*Ks*)	dN/dS	prot_ID [%]	cDNA_ID [%]	# non-syn var	# syn var
HARS	1536	579	37.7	181574	Nku-G-d-12e07.f1	413.8	0.0025	165.2	0.0251	0.09861	99.48	99.14	1	4
MTIF2	1992	309	15.5	161318	Nku-G-d-59b11.r1	236.9	0.0043	72.1	0.0417	0.10392	99.03	98.71	1	3
CYP11B2	933	524	56.2	15769	Nku-G-d-37d04.f1	391.4	0.0052	130.6	0.0484	0.10775	98.85	98.47	2	6
MLL3 (2 OF 2)	11544	609	5.3	175423	Nku-G-d-61c10.f1	453.9	0.0022	155.1	0.0198	0.11319	99.51	99.34	1	3
ZZZ3	2883	287	10.0	185460	Nku-G-d-20d12.f1	205.8	0.0051	76.2	0.0421	0.12168	98.94	98.58	1	3
ADAM8A	2148	463	21.6	208012	Nku-G-d-45e07.f1	339.4	0.0030	122.6	0.0237	0.12581	99.35	99.13	1	3
KLHL15	2115	1,042	49.3	13700	Nku-G-d-29h08.f1	819.1	0.0063	221.9	0.0489	0.12854	98.56	98.56	5	11
LAMB2L	6072	523	8.6	183309	Nku-G-d-63g03.r1	413.9	0.0050	108.1	0.0368	0.13479	98.85	98.85	2	4
EARS2	1536	621	40.4	12266	Nku-G-d-57h09.f1	391.9	0.0026	229.1	0.0180	0.14562	99.52	99.19	1	4
NFE2L3	1917	587	30.6	161585	Nku-G-d-23a08.r1	395.0	0.0052	190.0	0.0331	0.15721	98.97	98.63	2	6
MINK1	3786	373	9.9	2177	Nku-G-d-31f09.r1	261.3	0.0079	110.7	0.0473	0.16632	98.39	98.12	2	5
GTF2H2	1176	312	26.5	163318	Nku-G-d-50d10.f1	227.6	0.0045	84.4	0.0243	0.18343	99.04	99.04	1	2
SLC25A30	867	211	24.3	165726	Nku-G-d-48d09.f1	153.2	0.0067	56.8	0.0362	0.18409	98.57	98.57	1	2
ACOT11B	1725	455	26.4	193894	Nku-G-d-38d02.r1	348.4	0.0058	101.6	0.0313	0.18465	98.01	98.68	2	3
N4BP1	2811	817	29.1	161462	Nku-G-d-34e07.f1	640.0	0.0079	173.0	0.0425	0.18529	98.52	98.52	5	7
MBTPS1	3210	254	7.9	160773	Nku-G-d-18b10.f1	183.4	0.0056	68.6	0.0297	0.18873	98.81	98.81	1	2
ALG1	1383	277	20.0	17574	Nku-G-d-11f01.r1	200.1	0.0051	75.9	0.0271	0.18948	98.91	98.91	1	2
GPR155 (1 OF 2)	2421	346	14.3	175714	Nku-G-d-07d04.f1	246.2	0.0042	98.8	0.0213	0.19468	99.13	99.13	1	2
PPFIA2	2931	228	7.8	7500	Nku-G-d-02g05.r1	174.9	0.0118	53.1	0.0603	0.19576	97.37	97.81	2	3
MARK1	2385	323	13.5	743	Nku-G-d-38e01.r1	245.4	0.0082	75.6	0.0415	0.19706	98.13	98.44	2	3
Q0Q7C6_ORYLA	525	525	100.0	178389	Nku-G-d-33e08.f1	389.4	0.0079	132.6	0.0395	0.19931	98.29	98.29	3	5
ELOVL1B	1014	510	50.3	178228	Nku-G-d-10a08.f1	387.8	0.0053	122.2	0.0263	0.20354	98.82	99.02	2	3
CAMTA1 (1 OF 2)	2073	488	23.5	207535	Nku-G-d-38b01.f1	325.5	0.0094	160.5	0.0456	0.20583	98.15	97.94	3	7
RAD54L2	4440	629	14.2	159586	Nku-G-d-56e02.f1	429.3	0.0024	197.7	0.0103	0.23179	99.52	99.52	1	2
WDR4 (2 OF 2)	1164	369	31.7	27523	Nku-G-d-35h07.f1	295.5	0.0034	73.5	0.0142	0.23795	99.19	99.46	1	1
KAT7 (2 OF 2)	1878	360	19.2	3149	Nku-G-d-29g01.r1	267.3	0.0113	92.7	0.0460	0.24488	97.5	98.06	3	4
CR388231	2532	424	16.7	980	Nku-G-d-12g07.r1	284.6	0.0036	138.4	0.0146	0.24881	99.29	99.29	1	2
SQSTM1	765	286	37.4	8456	Nku-G-d-56d09.r1	220.7	0.0090	64.3	0.0343	0.26344	97.89	98.6	2	2
SLC30A9	1719	230	13.4	160549	Nku-G-d-16e09.f1	183.7	0.0059	44.3	0.0219	0.26799	98.68	99.12	1	1
GFOD1	1176	879	74.7	17839	Nku-G-d-20f11.f1	638.6	0.0159	237.4	0.0556	0.28533	96.58	97.49	10	13
UBAC1	1257	211	16.8	5031	Nku-G-d-18e01.f1	161.0	0.0063	49.0	0.0211	0.29645	98.57	99.05	1	1
ANKRD52 (2 OF 2)	5802	825	14.2	176361	Nku-G-d-42d08.f1	610.0	0.0033	215.0	0.0093	0.35721	99.27	99.52	2	2
PFKL (2 OF 2)	423	171	40.4	89104	Nku-G-d-37d10.f1	124.9	0.0165	46.1	0.0458	0.36005	96.49	97.66	2	2
CCNC	849	266	31.3	163424	Nku-G-d-37e06.f1	205.9	0.0000	58.1	0.0000	0.39739	100	100	0	0
SUSD1 (1 OF 2)	873	311	35.6	8850	Nku-G-d-06g07.f1	239.4	0.0126	69.6	0.0312	0.40540	97.09	98.38	3	2
MCM3AP	6330	1,043	16.5	175677	Nku-G-d-50h06.f1	725.5	0.0159	315.5	0.0388	0.40974	97.12	97.79	12	12
DSP (2 OF 2)	7038	323	4.6	192742	Nku-G-d-62e07.f1	242.9	0.0000	78.1	0.0000	0.41947	100	100	0	0
LMOD1 (2 OF 2)	3405	948	27.8	159365	Nku-G-d-42a11.f1	652.7	0.0031	295.3	0.0069	0.44353	99.37	99.58	2	2
STX17	876	189	21.6	163573	Nku-G-d-48a04.f1	155.1	0.0000	33.9	0.0000	0.44456	100	100	0	0
HGFA	1158	154	13.3	9211	Nku-G-d-56g07.r1	117.3	0.0000	35.7	0.0000	0.45730	100	100	0	0
EDC4	4164	280	6.7	193667	Nku-G-d-06d08.f1	221.7	0.0000	57.3	0.0000	0.46264	100	100	0	0
ATP2B3B	2847	229	8.0	2708	Nku-G-d-56a06.f1	199.2	0.0000	28.8	0.0000	0.46620	100	100	0	0
JMY (2 OF 2)	2595	606	23.4	175065	Nku-G-d-34h03.f1	415.9	0.0049	190.1	0.0104	0.46787	99.01	99.34	2	2
CPO	1053	209	19.8	10194	Nku-G-d-03d03.r1	142.3	0.0153	64.7	0.0321	0.47784	97.1	98.07	2	2
CARS2	1686	295	17.5	176993	Nku-G-d-51d12.f1	192.5	0.0109	101.5	0.0208	0.52136	97.96	98.64	2	2

Gene	length [bp] cds in Nofu	length nt-ali seq1 to seq2	[%]	GRZ transcript ID: Nofu_GRZ_cDNA_3_	Noku genom contig	N = avg no. non-syn sites	dN (*K*a)	S = avg no. syn sites	dS (*Ks*)	dN/dS	prot_ID [%]	cDNA_ID [%]	# non-syn var	# syn var
SH2D3A	1788	382	21.4	161217	Nku-G-d-04d02.f1	295.3	0.0137	85.7	0.0246	0.55693	96.85	98.43	4	2
RAPGEF3	2703	367	13.6	178504	Nku-G-d-19b05.r1	283.6	0.0071	82.4	0.0127	0.55888	98.36	99.18	2	1
C2CD3	6438	589	9.1	768	Nku-G-d-56e04.r1	424.9	0.0072	163.1	0.0125	0.57469	98.47	99.15	3	2
FDFT1	1251	345	27.6	25345	Nku-G-d-26e02.f1	281.1	0.0110	63.9	0.0166	0.65881	97.39	98.84	3	1
SEMA4G (1 OF 2)	2622	212	8.1	2365	Nku-G-d-29c08.r1	157.3	0.0130	52.7	0.0197	0.65931	97.14	98.57	2	1
SI:CH211-210C8	774	558	72.1	162668	Nku-G-d-05b05.f1	413.2	0.0050	144.8	0.0072	0.68591	98.92	99.46	2	1
CAPN5A	1911	535	28.0	8771	Nku-G-d-01a05.f1	427.2	0.0378	106.8	0.0545	0.69227	93.82	96.07	16	6
ZDBF2	654	256	39.1	59819	Nku-G-d-18g01.f1	207.2	0.0148	47.8	0.0211	0.70509	96.47	98.43	3	1
TLE4	1815	323	17.8	26055	Nku-G-d-22h05.f1	217.2	0.0141	103.8	0.0197	0.71466	97.2	98.44	3	2
SEC24C	3576	449	12.6	176336	Nku-G-d-38e07.f1	336.8	0.0092	110.2	0.0091	1.00503	97.99	99.11	3	1
EVC2	3825	264	6.9	175757	Nku-G-d-58g02.f1	171.2	0.0126	89.8	0.0107	1.17755	96.59	98.48	2	1
MPHOSPH9	3333	267	8.0	3343	Nku-G-d-11a06.f1	172.1	0.0269	94.9	0.0209	1.28390	95.51	97.75	5	2
CIAPIN1	945	249	26.3	39469	Nku-G-d-47h03.r1	174.4	0.0232	74.6	0.0142	1.63537	95.18	97.99	4	1
ATAD5A	5187	971	18.7	159992	Nku-G-d-01b05.f1	676.7	0.0090	289.3	0.0035	2.59650	98.14	99.28	6	1
BAIAP2L1A	1449	207	14.3	160850	Nku-G-d-22c01.r1	184.3	0.0000	22.7	0.0000	n.a.	100	100	0	0
UNC93B1	1818	247	13.6	194220	Nku-G-d-17f12.f1	191.8	0.0106	54.2	0.0001	n.a.	97.56	99.19	2	0
SF1	1770	156	8.8	9907	Nku-G-d-06e03.f1	122.7	0.0082	33.3	0.0001	n.a.	98.08	99.36	1	0
KDM2AA	3708	275	7.4	175268	Nku-G-d-47d07.r1	223.6	0.0044	49.4	0.0000	n.a.	98.9	99.63	1	0
TBK1	2166	302	13.9	9307	Nku-G-d-06b06.f1	214.0	0.0095	86.0	0.0001	n.a.	98	99.33	2	0
LRRC34	543	195	35.9	72639	Nku-G-d-12b01.f1	131.8	0.0273	63.2	0.0003	n.a.	95.38	98.46	4	0
SERINC1	1389	262	18.9	33551	Nku-G-d-58c11.f1	218.5	0.0000	42.5	0.0000	n.a.	100	100	0	0
HDX	2124	276	13.0	193833	Nku-G-d-14c07.f1	210.1	0.0141	65.9	0.0001	n.a.	96.74	98.91	3	0
ANKRD27	2676	294	11.0	207471	Nku-G-d-47b11.r1	202.9	0.0052	91.1	0.0001	n.a.	98.98	99.66	1	0
MPP4 (2 OF 3)	1893	349	18.4	177159	Nku-G-d-19e05.f1	278.7	0.0036	69.3	0.0000	n.a.	99.14	99.71	1	0
FRMD1	1575	282	17.9	161730	Nku-G-d-36c11.f1	189.1	0.0162	92.9	0.0002	n.a.	96.81	98.94	3	0
**median**	**1,992**	**323**	**17**			**243**	**0.0036**	**85**	**0.0211**	**0.1317**	**99.15**	**99.11**	**1.0184**	**2.0567**
**total**	**306528**	**44710**				**32976**		**11592**					**177**	**298**
