# Supplementary material for: Parallel evolution of senescence in annual fishes in response to extrinsic mortality
Source: BMC Evol Biol. 2013 Apr 3;13:77. doi: 10.1186/1471-2148-13-77 (PMC3623659; doi:10.1186/1471-2148-13-77)
Supplement: Additional file 12: Table S1 — The number of dataloggers which were still submerged (water) and loggers exposed on dry bottom (dry) recovered at each region. [file 1471-2148-13-77-S12.docx]

**Table S1.** The number of dataloggers which were still submerged (water) and loggers exposed on dry bottom (dry) recovered at each region

|  | 2011 |  |  |  | 2012 |  |  |  |
| --- | --- | --- | --- | --- | --- | --- | --- | --- |
|  | water |  | dry |  | water |  | dry |  |
| region | N | % | N | % | N | % | N | % |
| humid | 2 | **100** | 0 | **0** | 3 | **75** | 1 | **25** |
| semi-dry | 1 | **17** | 6 | **83** | 2 | **17** | 10 | **83** |
